# Evidence for disseminated tumor cells in lymphatic vessels afferent to sentinel lymph nodes in patients diagnosed with cervical cancer

**DOI:** 10.1002/cnr2.1366

**Published:** 2021-03-14

**Authors:** Shadi Younes, Andreas M. Kaufmann, Norman Häfner, Katrin Beer, Lars Jansen, Juliane Sanft, Gita Mall, Susan Koops, Matthias Dürst, Achim Schneider

**Affiliations:** ^1^ Department of Gynaecology Klinikum Bremen‐Nord Bremen Germany; ^2^ Department of Gynaecology Charité Medical University Berlin Berlin Germany; ^3^ Department of Gynaecology Jena University Hospital Jena Germany; ^4^ Institute for Forensic Medicine Jena University Hospital Jena Germany; ^5^ Institute for Cytology and Dysplasia, MVZ im Fürstenberg‐Karree Berlin Germany

**Keywords:** afferent lymphatic vessels, HPV oncogene transcripts, sentinel lymph node

## Abstract

**Background:**

In patients diagnosed with cervical cancer, the purpose of lymphadenectomy is the removal of lymph nodes for diagnosis and potential treatment of metastasized tumor cells. It is unclear if afferent lymphatic vessels harbor tumor cells and, thus, may pose additional risk for recurrence or progression if not removed.

**Aim:**

In this feasibility study, we analyzed the lymphatic vessels afferent to sentinel lymph node (SLN) using a highly sensitive and specific molecular marker for cervical cancer cells.

**Methods and Results:**

Twenty patients diagnosed with cervical cancer of FIGO stage IA1 to IIB2 underwent laparoscopic SLN removal. Labeling was done using patent blue and the afferent lymphatic vessels were harvested from the parametric tissue and frozen at −80°C. HPV DNA type was evaluated in the primary tumor. Lymphatic vessels afferent to the sentinel lymph nodes were analyzed for the presence of viral oncogene transcripts of the respective HPV type. In one of 18 patients, all with tumor stage ≤IBI and pN0 by conventional histopathology, HPV mRNA could be detected in two of four lymphatic vessels, whereas at least one of the lymphatic vessel biopsies of both patients with tumors ˃4 cm and pN1 status was HPV mRNA positive. No clinical correlation with recurrence after a median follow‐up of 9 years was noticed.

**Conclusion:**

HPV mRNA indicative of disseminated tumor cells could be detected in lymphatic vessels. The relevance of harvesting lymphatic vessels afferent to SLN in order to increase oncologic safety will have to be investigated in a future prospective study.

**Abbreviations**
cDNA: complementary DNA
CxCa: invasive cervical carcinoma
HE: hematoxylin‐eosin
ICG: indocyanine green
qRT‐PCR: quantitative real‐time PCR
STR: short tandem repeats

## 1 INTRODUCTION

In patients with cervical cancer, metastatic spread to lymph nodes is the most important prognostic marker and is decisive for the selection of therapy. Histopathological examination is standard medical care for the past 100 years. In order to decrease morbidity associated with systematic lymphadenectomy, sentinel lymph node removal is accepted for women with low volume tumors.^1^ To avoid false‐negative results associated with selective bilateral pelvic sentinel lymph node harvesting, ultra‐staging was investigated in order to identify micrometastasis and isolated tumor cells. Immunohistochemical staging for cytokeratin markers is simple and convenient^2^ but its clinical predictive value is controversially discussed.^3^, ^4^, ^5^ In terms of molecular markers, it was shown that HPV type specific mRNA (viral oncogene transcripts) is not only more specific than cytokeratin19 mRNA for the detection of tumor cells but is also of prognostic significance in patients with pN0 status.^6^, ^7^

Several studies have recently been initiated to reduce radicality of treatment where only sentinel lymph nodes and no parametria are removed,^8^, ^9^, ^10^ leaving afferent lymphatic vessels accompanied with minute lymph nodes in situ. Tracers such as patent blue or infrared fluorescence from indocyanine green^11^, ^12^ allow identification of small lymphatic vessels, whereas radioactive labeling is only suitable for lymph node identification alone.

Selective removal of SLN without the afferent lymphatic vessels and/or preservation of the parametria, which contain the afferent vessels, may lead to the risk that tumor cell containing lymphatic vessels remain in situ and may lead to loco‐regional recurrence. In our study, we address the following questions:

•Do lymphatic vessels afferent to the sentinel lymph node contain micrometastasis or isolated tumor cells?

•Does presence or absence of tumor cells in lymphatic vessels correlate with the histopathologic status of the respective SLN?

## 2 MATERIALS AND METHODS

### 2.1 Patients

In this monocentric prospective study, 20 patients (aged 26‐47 years, median 33.5) with stage IA1 to IIB2 cervical cancer predominantly of squamous cell histologic type were included (Table 1). Fourteen patients were treated by applying the sentinel concept concurrent with surgical removal of the tumor. For three further patients, beside SLN detection, complete lymphadenectomy (LNE) was performed concurrent with tumor surgery. For the remaining three patients, only pelvic LNE or pelvic and para‐aortic LNE staging was conducted. Sentinel lymph nodes were identified by laparoscopy using patent blue as a tracer. In our cohort, two patients with tumors ˃4 cm were included because of the high likelihood to detect HPV mRNA in lymphatic vessel biopsies. Of each patient, at least one tissue sample comprising afferent lymphatic vessels (ie, leading from the primary tumor to the SLN) was removed and shock frozen at −80°C for subsequent molecular analysis. For one of the two patients with large tumors, no SLN could be detected, instead one lymphatic vessel biopsy afferent to a lymph node and a lymph node biopsy were taken. After surgery, all lymph nodes were paraffin‐embedded and examined by standard histologic examination. Ultra‐staging of SLN was not performed.

**TABLE 1:** Clinical parameters of patients, SLN location, and lymph vessels characteristics

Patient ID #	Histologic type	HPV type in primary tumor	Age at surgery	Surgery	TNM code	Number of SLN	Lymph vessel biopsy	Biopsy #	Lymph vessel histology	HPV mRNA
1	Villoglandular adeno‐ca	16	34	Staging pelvic and para‐aortic LNE	pT1a1pN0(0/29)G2R0L0V0	2 left, 5 right	Pelvic left	2963	LV	
2	Squamous cell ca	16	27	SLN and RVT	pT1b1pN0(0/8)G2R0L1V0	6 left, 1 right	Pelvic left	2678	LN, LV	
							Pelvic right	2692	LV	
3	Squamous cell ca	16	33	SLN and RVT	pT1a1pN0(0/2)G2R0L0V0	1 left, 1 right	Pelvic left	2635	LV	
							Pelvic right	2636	LV	
4	Squamous cell ca	16	42	SLN and VALRH	pT1b1pN0(0/4)G2R0L0V0	2 left, 1 right	Pelvic left	2658	LV	
							Pelvic right	2653	LV	
5	Clear cell adeno‐ca	16	26	SLN and RVT	pT1b1pN0(0/6)G2R0L0V0	3 left, 3 right	Pelvic left	2829	LV	
							Pelvic right	2830	LV	
6	Lymphoepithelial‐like ca	18	33	Pelvic LNE and RVT	pT1b1pN0(0/26)G3R0L0V0	4 left, 2 right	Pelvic left	2827	LV	
							Pelvic left	2828	LV	
7	Squamous cell ca	18	32	SLN and RVT	pT1a2pN0(0/3)L0V0R0	1 left, 2 right	Pelvic left	2914	LV	
							Pelvic right (1)	2904	LN, LV	
							Pelvic right (2)	2915	LV	
							Pelvic right (2)	2916	LV	
8	Adeno‐ca	16	40	SLN and VALRH	pT1b1pN0(0/2)G2L1V0	1 left, 1 right	Pelvic left	2922	LV	
9	Squamous cell ca	16	34	Staging pelvic and para‐aortic LNE	pT2b2pN1(35/44)M1a(10/24 para‐aortic and supraclavicular)	Not detected	Pelvic left^a^	2874	LV	E6*I 3/6; E7 5/6
							Pelvic LN right^b^	2884	LN, Metastases, LV	E6*I 6/6; E7 6/6
10	Squamous cell ca	16	27	SLN and re‐conization	pT1a1pN0(0/2)R0L1V0	1 left, 1 right	Pelvic left	2847	LV	
							Pelvic right	2848	LV	
11	Squamous cell ca	16	33	SLN and RVT	pT1b1pN0(0/9)G2R0L1V0	7 left, 2 right	Pelvic left	3019	LV	
							Pelvic right	3020	LV	
12	Adeno‐ca	16	36	Staging pelvic and para‐aortic LNE	pT2a2pN1(2/11)cM0G3 (0/32 para‐aortic)	3 right	Pelvic right	2858	LV	E6*I 1/6; E7 4/6
							Para‐aortic	2857	LV	
							Para‐aortic	2856	LV	
13	Squamous cell ca	16	26	SLN and RVT	pT1a2N0(0/1)G3L1V0R0	1 right	Pelvic right	2879	LN, LV	
14	Squamous cell ca	16	27	SLN and RVT	pT1b1pN0(0/2)G3R0L0V0	1 left, 1 right	Pelvic left	2854	LN, LV, endosalpingiosis	
							Pelvic right	2855	LN, LV	
15	Squamous cell ca	16	34	Pelvic LNE and VALRH	pT1b1pN0(0/43)L1V0R0	1 left, 3 right	Pelvic left	2793		
							Pelvic right	2792	LV	
16	Squamous cell ca	16	47	Pelvic LNE and VALRH	pT1a1pN0(0/18)G3R0L0V0	5 left, 4 right	Pelvic left	2824	LN, LV	
							Pelvic right	2832	LN, LV	
17	Squamous cell ca	16	38	SLN and RVT	pT1a2pN0(0/3)M0G2L0V0R0	2 left, 1 right	Pelvic left	2861	LV	
							Pelvic right	2860	LV	
18	Adenosquamous ca	16	28	SLN and RVT	pT1a1N0(0/2)G2L1V0	1 left, 1 right	Pelvic right	2859	LV	
19	Squamous cell ca	16	45	SLN and VALRH	pT1a2pN0(0/3)G2R0L0V0	1 left, 2 right	Pelvic left	2660	LV	
							Pelvic right (1)	2657	LV	E6*I 1/6; E7 3/6
							Pelvic right (2)	2661	LN, LV	E6*I 3/6; E7 5/6
							Pelvic right (2)	2662	LV, thrombosis	
20	Squamous cell ca	16	34	SLN and RVT	pT1b1pN0(0/5)G2R0L0V0	4 left, 1 right	Pelvic right	2938	LV	

Abbreviations: LN, lymph node; LNE, lymphadenectomy; LV, lymphatic vessel; RVT, radical vaginal trachelectomy; SLN, sentinel lymph node; VALRH, vaginal‐assisted laparoscopic radical hysterectomy.

^a^ Lymph vessel leading to an unstained node.

^b^ Instead of a lymph vessel biopsy, a lymph node biopsy was analyzed.

### 2.2 HPV‐genotyping of primary tumors

HPV‐genotyping was performed using genomic DNA extracted from FFPE primary tumors. Thirty‐seven different genital HPV types, including all hrHPV types, can be amplified by the GP5+ and GP6+ bio assay.^13^

### 2.3 STR analysis

Sample identity of all primary cancers and their corresponding lymph vessel biopsies were confirmed by STR analyses. The standard ESS DNA profiles (amelogenin, vWA, SE33, THO1, D21S11, D8S1179, D3S1358, FGA, D18S51, D16S539, D19S433, D1S1656, D2S441, D2S1338, D10S1248, D12S391, and D22S1045) were obtained by amplification with the multiplex PCR Kits Powerplex ESX17 Fast (Promega) and ESSPlexSE QS (Qiagen) following the manufacturer's instructions with reduced PCR volume of 12.5 μL employing 1 ng DNA. A positive control (corresponding to the respective multiplex kits) and a negative control (sterile water) were analyzed in each amplification. All amplifications were done in a T3000 Thermal Cycler (Biometra, Göttingen, Germany). Amplification products were separated and detected on the ABI3500 Genetic Analyzer (ThermoFisher). Alleles were assigned in comparison to the corresponding allelic ladders. Electrophoresis results were analyzed with GeneMapper ID‐X Software. Allele peaks were interpreted when greater than or equal to 100 RFUs.

### 2.4 Serial sectioning of lymphatic vessel biopsies for hematoxylin‐eosin staining, immunohistochemical analyses, and RNA extraction

Frozen tissue samples of lymphatic vessels afferent to the sentinel nodes were carefully embedded in TissueTec for sectioning. All steps were conducted in a HPV‐free laboratory to avoid contamination. Serial tissue sections (7 μm) were prepared of which the first and last sections were hematoxylin‐eosin (HE) stained. The flanking sections were used for immunohistochemical staining of cytokeratins (AE1/3) and podoplanin (D2‐40). RNA was extracted from 20 to 30 consecutive sections (10 μm) flanked by the sections for immunohistochemistry using NucleoSpin RNA Plus XS kit (Machery‐Nagel) according to the manufacturer's recommendation, which also included DNase treatment. An ovarian cancer biopsy was sectioned and processed in the same way at intervals of five lymphatic samples and served as negative control.

### 2.5 Detection of HPV oncogene transcripts in lymphatic vessels

#### 2.5.1 cDNA synthesis

RNA concentration and quality were determined by spectrophotometry (NanoDrop ND‐1000) and gel electrophoresis. Up to 500 ng of total RNA was reverse transcribed in a 20 μL reaction comprising 20 pmole CDS primer (5′‐T_30_VN‐3′), 0.5 mM of each dNTP, 50 mM Tris‐HCl (pH 8.3), 75 mM KCl, 3 mM MgCl_2_ 10 mM DTT, 20 U RNaseOUT, and 100 U SuperScript II Reverse Transcriptase (ThermoFisher Scientific). To allow optimal annealing, primer and RNA were incubated at 70°C for 10 minutes and quick chilled on ice. The remaining reagents were then added in the form of a master mix and cDNA synthesis was done at 42°C for 1 hour. The reaction was stopped at 70°C for 15 minutes and stored at −80°C until PCR analyses. A second round of cDNA synthesis was done with a hexanucleotide primer (50 pmole per reaction) instead of the CDS primer.

#### 2.5.2 Quantitative PCR

Quantitative PCRs (qPCRs) were performed in triplicate runs with assays designed to detect GAPDH and E6*I‐E7 transcripts. For the detection of E6*I‐E7 transcripts, mRNA primer pairs for E6*I and E7, respectively, were used (Table 2). Both CDS‐ and hexanucleotide‐primed cDNAs were analyzed, thereby providing for each sample six PCR results per target. The reactions consisted of 10 μL Fast Start Universal SYBRGreen‐Master Mix (Roche Applied Science, Mannheim, Germany), 5 pmole forward and reverse primer each, 2 μL cDNA template, and was adjusted to 20 μL with aqua dest. Cycling included a 10 minutes initial denaturation step at 98°C, 40 cycles of 15 seconds denaturation (98°C), 20 seconds annealing (60°C), and 40 seconds elongation (72°C) and 10 minutes final elongation step (72°C). The melting temperature of the PCR product was determined to ensure specificity.

**TABLE 2:** Primers used for qRT‐PCR

Gene	GenBank	Sequence 5′‐3′		Size (bp)	Ta (°C)
*HPV16*	K02718.1				
E6*I	nt 103‐427^a^	F	AATGTTTCAGGACCCACAGG	143	57
		R	CTTTTGACAGTTAATACACCTCACG		
E7	nt 563‐666	F	TGCATGGAGATACACCTACATTG	104	57
		R	CTCCTCCTCTGAGCTGTCATTTA		
*HPV18*	AY262282.1				
E6*I	nt 120‐426^a^	F	GATCCAACACGGCGAC	125	57
		R	ACCGCAGGCACCTCT		
E7	nt 751‐841	F	CGAACCACAACGTCACACA	91	57
		R	TCGAAGGTCGTCTGCTGAG		
*GAPDH*	M33197.1	F	GCGACACCCACTCCTCCACC	119	57
	nt 923‐1041	R	GAGGTCCACCACCCTGTTGC		

Abbreviations: F, forward; R, reverse; Ta, annealing temperature.

^a^ E6*I splice HPV16 nt 226‐409 and HPV18 nt 234‐415.

All samples were analyzed for the presence of E6*I‐E7 mRNA of HPV16 and HPV18, respectively. By this approach, the samples from patients with HPV16 positive tumors served as negative controls for the HPV18 assays and vice versa.

#### 2.5.3 Algorithms for HPV mRNA scoring

A previous study has shown that the qPCR assays applied here have an analytical sensitivity of one tumor cell equivalent in a background of 10^5^ HPV‐negative HaCaT cells.^6^ To account for stochastic effects in samples with low template numbers, we conducted triplicate qPCR runs using two independently generated cDNAs. To be scored positive for HPV mRNA at least one of six reactions, both for E6*I and E7, respectively, had to be positive. These requirements are based on the fact that the most abundant HPV mRNA species in cervical cancers is polycistronic and comprises a spliced E6 open reading frame (E6*I), E7 and further downstream viral genes.^6^, ^14^, ^15^ Moreover, the incorporation of a splice‐specific PCR (E6*I) excluded the detection of contaminating viral DNA. Although the Ct values for GAPDH ranged from 19 to 29, all samples were scored for the presence of HPV mRNA.

### 2.6 Immunohistochemical staining for multi‐cytokeratins (AE1/AE3) and podoplanin (D2‐40)

In order to assess the histology of the lymph vessel biopsies, sections flanking the tissue for RNA extraction were stained for the epithelial cells (AE1/3) and lymphatic cells (D2‐40). Briefly, cryosections of 7 μm thickness were fixed on Superfrost Plus slides with 4% paraformaldehyde for 10 minutes. Slides were washed with tris‐buffered saline (50 mM Tris, 150 mM NaCl) and 0.1% Tween‐20 (TBST) and incubated in 0.6% H_2_O_2_ for 7 minutes. Staining was performed for 1 hour at room temperature with AE1/3 (Dako) diluted to a final concentration of 1.7 μg/mL and podoplanin D2‐40 (Dako) diluted to a final concentration of 0.5 μg/mL. For detection, the DAKO EnVision System was used according to the manufacturer's protocol. Finally, slides were counterstained with hematoxylin, dehydrated in alcohol, equilibrated in xylol, and embedded using RotiHistokitt (Roth).

## 3 RESULTS

### 3.1 Histological assessment of lymphatic vessels biopsies

**FIGURE 1:**
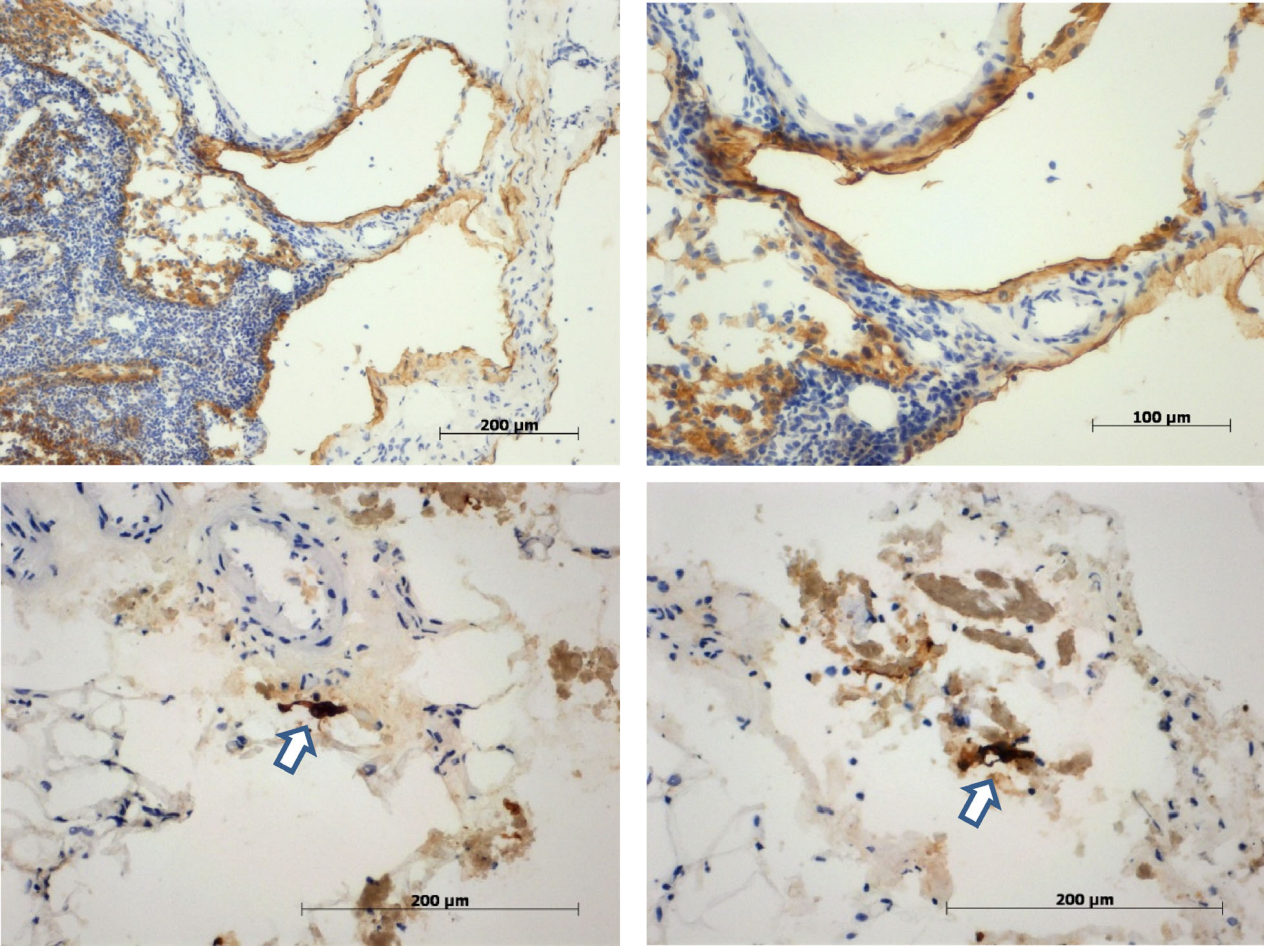
*Top row*: Biopsy #2678 of patient 2 comprising a small lymph node and podoplanin stained lymphatic vessels (×10 and ×20). *Bottom row*: Lymphatic vessels showing AE1/3 stained tumour cells (arrows) at two different sites in biopsy #2874 of patient 9 (×40)

The presence of lymphatic vessels could be confirmed in HE‐stained sections in all but one biopsy (Figure 1). In most biopsies, varying amounts of fat, connective tissue, blood vessels, and nerve cells were also evident. Moreover, in 10 biopsies, small lymph nodes (≤1 mm) were also detected (Table 1 and Figure 1). One biopsy comprised a lymph node with metastatic disease.

### 3.2 Detection of HPV oncogene transcripts

Four of 33 lymphatic vessel biopsies of 18 patients with HPV16 positive primary tumors were positive for low levels of HPV16 E6*I‐E7 oncogene transcripts. Ct values were in the range of 35 to 39, and multiple testing revealed stochastic effects characteristic for the detection of single tumor cells (see definition of HPV mRNA positivity in Section 2). As expected, the lymph node with metastases (patient #9) revealed high levels of oncogene transcripts (Ct value 19). For this biopsy, transcript concentration was 4‐fold higher than that detected in an equivalent amount of cDNA derived from HPV16‐positive SiHa cells. All six lymphatic vessel biopsies from two patients with HPV18 positive primary tumors were negative for HPV16 mRNA. This was also the case for all ovarian cancer control sections prepared intermittently when sectioning the lymphatic vessel biopsies. None of the biopsies were positive for HPV18 oncogene transcripts. HPV18‐positive C4‐I cells served as positive control. Repeated dilution experiments of RNA derived from HPV‐positive SiHa and C4‐I cells in a background of RNA from 5 × 10^4^ HPV‐negative HaCaT cells allowed the reliable detection of HPV oncogene transcripts corresponding to a 1 to 2 SiHa or C4‐I cells per PCR reaction.

### 3.3 Correlation between HPV‐positive lymphatic vessels and the histopathology of the corresponding lymph nodes

With exception of two cases (ID #9 and #12 in Table 1), all patients were pN0. One lymphatic vessel biopsy of each patient with pN1 status contained HPV16 oncogene transcripts. In another patient, two of four lymphatic vessel biopsies were HPV mRNA positive despite pN0 status (ID #19, Table 1). The details of the above three cases are as follows:

ID #9: The only patient in this cohort without identifiable sentinel lymph nodes. Thirty‐five of 44 lymph nodes were positive for metastasis by final histopathological assessment. In this patient, beside one lymphatic vessel biopsy, a lymph node biopsy was also taken for HPV mRNA analyses. Both biopsies were taken from different sites. The lymph node (left) contained high levels, and the lymphatic vessel (right) contained low levels of oncogene transcripts, respectively.

ID #12: Three unilateral sentinel lymph nodes on the right side were free of metastasis by final histopathological assessment. Two of 11 pelvic lymph nodes on the left side were positive by histopathology. Besides, 19 left‐sided para‐aortic lymph nodes and 13 right‐sided para‐aortic lymph nodes were negative. The lymphatic vessel biopsy afferent to one of the right sentinel nodes contained low levels of oncogene transcripts. Two further para‐aortic lymph vessel biopsies were HPV mRNA negative.

ID #19: Overall, three pelvic sentinel lymph nodes, one on the left, two on the right side, were detected. There was no evidence of metastasis by final histopathological assessment. Two of three lymphatic vessel biopsies afferent to the right sentinel nodes contained low levels of oncogene transcripts. The lymphatic vessel on the left side was HPV mRNA negative.

Overall, the presence of low‐level HPV oncogene transcripts in lymphatic vessels does not correlate with the histopathology of the corresponding sentinel lymph nodes.

### 3.4 Comparison between HPV mRNA status of lymphatic vessels and AE1/3 staining

RNA extracted from 20 to 30 consecutive tissue sections was used for HPV mRNA analyses. Sections flanking the tissue for RNA extraction were used for HE staining and immunohistochemical staining for cytokeratins (AE1/3). The only biopsy (#2884, patient 9) with a high level of HPV16 oncogene transcripts was taken from a lymph node biopsy harboring a metastasis. In four lymphatic vessel biopsies, only low levels of HPV16 oncogene transcripts indicative of single tumor cells could be detected. For one of these biopsies (#2874, patient 9) single AE1/3 positive cells could be detected albeit not clearly within a lymphatic vessel (Figure 1). Two other biopsies (#2854, patient 14 and #2938, patient 20) with single AE1/3 positive cells were negative for HPV mRNA. Of note is that the AE1/3 positive cells in #2854 probably resulted from endosalpingiosis. Overall, the number of lymph vessel biopsies is too small for a valid comparison of mRNA detection and immunohistochemical staining.

## 4 DISCUSSION

The presence of circulating tumor cells in blood is an independent predictive factor in cancer, for example, breast, prostate, colorectal,^16^ and locally advanced cervical cancer of patients undergoing primary chemoradiation.^17^ However, early stage cervical cancer metastasizes exclusively via lymphatic vessels and circulation of tumor cells in the bloodstream appears as a late event in women diagnosed with cervical cancer.^18^ Histopathological detection of tumor cells in lymphatic vessels of the tumor (L1) is associated with the presence of lymph node metastasis and, thus, a worse prognosis for the patient.^19^ Therefore, the question must be asked if removal of lymphatic vessels can be of diagnostic and potentially therapeutic value? This becomes even more important since the introduction of targeted sentinel lymph node harvesting and disregarding systematic lymphadenectomy and omitting connective tissue close to the uterus in situ, which contains lymphatic vessels and small lymph nodes. There is no previous report on the presence of molecularly detected tumor cells in lymphatic vessels associated with the sentinel lymph node. We show that in the case of one of 18 patients, all with tumor stage ≤IBI and pN0 status by conventional histopathology, two of four lymphatic vessels leading to the sentinel lymph node were HPV mRNA positive. It may thus be postulated that lymphatic vessels should also be harvested together with the sentinel node to decrease the risk of leaving viable tumor cells behind, especially when no adjuvant therapy is planned. Since only color staining allows the detection of lymphatic vessels, a radioactive marker alone is suboptimal for the implementation of the sentinel concept and if used should be combined with a color tracer such as patent blue and/or ICG.

Somewhat unexpected is the observation that three lymphatic vessel biopsies afferent to sentinel nodes harbored HPV mRNA although the sentinel nodes were free of metastasis by histopathology (ID #12 and 19 in Table 1). The most likely explanation for this phenomenon is that micro‐metastases were missed by conventional histopathology.

In two patients (ID #12 and #13), sentinel lymph nodes were detected only unilaterally, and in one patient (ID #9) detection failed completely. This finding is not uncommon for patients with tumors ˃4 cm (patients #9 and #12) and is attributed to the presence of tumor cells blocking the spread of the dye.^1^

It should be emphasized that viral oncogene transcripts are highly specific molecular markers for the detection of viable tumor cells. The constitutive expression of viral E6 and E7 genes is required for the maintenance of the transformed phenotype.^20^ In the context of lymph nodes or lymphatic vessels, the detection of HPV DNA would not allow to discriminate between viable tumor cells and fragmented viral DNA in lymphocytes or its presence in interstitial fluid. In two recent studies, using commercially available kits, 4 out of 125 (3.6%) and 9 out of 134 (8%) histologically free lymph nodes were positive for viral oncogene transcripts.^21^, ^22^ These observations are in line with previous studies^6^, ^7^ and underscore the high diagnostic potential of viral oncogene transcripts.

Due to the small number of patients and the surgical resection of afferent vessels in our study, we cannot hypothesize if removal of tumor cell containing lymphatic vessels is of therapeutic value. In the three patients with positive lymphatic vessels no recurrence of tumor was observed after more than 5 years follow‐up. Only 1 out of 20 patients (ID# 6) recurred locally, underwent surgery and chemoradiation, and is free of disease with a follow‐up of more than 6 years: her lymphatics were free of HPV mRNA.

## 5 CONCLUSION

HPV mRNA indicative of disseminated tumor cells could be detected in lymphatic vessels. The relevance of harvesting lymphatic vessels afferent to SLN in order to increase oncologic safety will have to be investigated in a future prospective study. Moreover, in such a study, the tissue sections, which would normally be discarded during ultra‐staging of SLN, could be used to determine HPV mRNA levels and would thus also permit a direct comparison of both approaches.

### 5.1 Limitations

Although the primary aim of this study was to provide evidence for disseminated tumor cells in lymphatic vessels, ultra‐staging of the respective SLN would have provided valuable additional information. Clearly, routine histopathological examination of SLN is not sensitive enough to detect micro‐metastases or small tumor cell clusters. This aspect will be kept in mind for conception of a subsequent study.

## AUTHOR CONTRIBUTIONS

**Shadi Younes:** Investigation; resources. **Andreas M Kaufmann:** Data curation; formal analysis; investigation. **Norman Häfner:** Formal analysis; methodology. **Katrin Beer:** Investigation; methodology. **Lars Jansen:** Investigation; methodology. **Juliane Sanft:** Methodology; validation. **Gitta Mall:** Methodology; validation. **Susan Koops:** Investigation; visualization. **Matthias Dürst:** Conceptualization; formal analysis; supervision; writing‐original draft; writing‐review & editing. **Achim Schneider:** Conceptualization; formal analysis; supervision; writing‐original draft; writing‐review & editing.

## CONFLICT OF INTEREST

The authors have no disclosures and declare to have no conflicts of interests concerning this manuscript.

## ETHICAL STATEMENT

This study was approved by the Ethics Committee of Charité (No. EA4/132/11) and the patient samples obtained and data used were in accordance with German medical council regulations. Informed written consent was obtained from all participants.

## Data Availability

Further data that support the findings of this study are available from the corresponding author upon reasonable request.
